# Nonindigenous Plant Advantage in Native and Exotic Australian Grasses under Experimental Drought, Warming, and Atmospheric CO_2_ Enrichment

**DOI:** 10.3390/biology2020481

**Published:** 2013-03-27

**Authors:** Robert C. Godfree, Bruce C. Robertson, Washington J. Gapare, Miloš Ivković, David J. Marshall, Brendan J. Lepschi, Alexander B. Zwart

**Affiliations:** CSIRO Plant Industry, GPO Box 1600, Canberra, ACT 2601, Australia; E-Mails: Bruce.Robertson@csiro.au (B.C.R.); Washington.Gapare@csiro.au (W.J.G.); Milos.Ivkovich@csiro.au (M.I.); David.Marshall@csiro.au (D.J.M.); Brendan.Lepschi@csiro.au (B.J.L.); Alec.Zwart@csiro.au (A.B.Z.)

**Keywords:** invasive species, climate change, extreme climatic events, drought, adaptation, plasticity, CO_2_, warming, *Nassella neesiana*, nonindigenous advantage, open top chamber

## Abstract

A general prediction of ecological theory is that climate change will favor invasive nonindigenous plant species (NIPS) over native species. However, the relative fitness advantage enjoyed by NIPS is often affected by resource limitation and potentially by extreme climatic events such as drought. Genetic constraints may also limit the ability of NIPS to adapt to changing climatic conditions. In this study, we investigated evidence for potential NIPS advantage under climate change in two sympatric perennial stipoid grasses from southeast Australia, the NIPS *Nassella neesiana* and the native *Austrostipa bigeniculata*. We compared the growth and reproduction of both species under current and year 2050 drought, temperature and CO_2_ regimes in a multifactor outdoor climate simulation experiment, hypothesizing that NIPS advantage would be higher under more favorable growing conditions. We also compared the quantitative variation and heritability of growth traits in populations of both species collected along a 200 km climatic transect. In contrast to our hypothesis we found that the NIPS *N. neesiana* was less responsive than *A. bigeniculata* to winter warming but maintained higher reproductive output during spring drought. However, overall tussock expansion was far more rapid in *N. neesiana*, and so it maintained an overall fitness advantage over *A. bigeniculata* in all climate regimes. *N. neesiana* also exhibited similar or lower quantitative variation and growth trait heritability than *A. bigeniculata* within populations but greater variability among populations, probably reflecting a complex past introduction history. We found some evidence that additional spring warmth increases the impact of drought on reproduction but not that elevated atmospheric CO_2_ ameliorates drought severity. Overall, we conclude that NIPS advantage under climate change may be limited by a lack of responsiveness to key climatic drivers, reduced genetic variability in range-edge populations, and complex drought-CO_2_ interactions.

## 1. Introduction

Over the past decade, there has been growing recognition that the invasion of plant communities by nonindigenous plant species (NIPS) is likely to interact synergistically with other drivers of environmental change to fundamentally alter global ecosystem processes [1,2]. With now overwhelming evidence that anthropogenic CO_2_ emissions are altering the earth’s climate [3,4], a key concern is whether climate change is likely to differentially alter the fitness of native and nonindigenous plant species. Developing a level of knowledge sufficient to answer this question with any degree of certainty or broad applicability is likely to be an extremely challenging task, especially given the traditional difficulty in predicting the success of invasive species generally [5]. Understanding the links between the traits and attributes of NIPS and key climatic drivers will be an important step towards achieving this goal [6] and will underpin our ability to manage or avoid the detrimental impacts of NIPS in the coming century.

It has long been argued that climate change is likely to favor invasive species and increase their impacts on recipient ecosystems [1,5]. This is due in large part to the fact that many NIPS share advantageous traits that, in addition to increasing general invasiveness, are also likely to provide a fitness advantage under new or changing climatic regimes. For example, many invasive NIPS have broad geographic ranges or environmental niches [1,7], display high levels of phenotypic plasticity [8,9,10], and have short generation times or morphological features that facilitate dispersal [1,11]. Many also exhibit ecologically important traits that increase fitness relative to native species [12,13], and often, but not always, respond more strongly to atmospheric CO_2_ enrichment [14,15,16]. NIPS also frequently undergo rapid adaptive evolution for fitness-enhancing traits [17,18], even following population bottlenecks [19], and clinal variation in climate-related traits has been widely observed among invasive plant species (e.g., [20,21,22,23,24]).

On the other hand, it is clear on both ecological and evolutionary grounds that increasing out-performance of native species by NIPS under climate change is far from a *fait accompli*. A key factor determining the fitness of many invasive species is suitability of the abiotic environment, and the relative growth and competitive ability of NIPS, both seen as important drivers of invasiveness [25,26], is often lower than that of native species in resource-limited environments [27]. Links between increased resource availability, plant invasiveness and disturbance (especially in *r*-selected species; [28]) indicate that the response of NIPS to climate change will depend strongly on concurrent changes in other anthropogenic pressures [6,29]. Genetic factors can also constrain the evolutionary capacity of NIPS, including fitness tradeoffs between traits [30,31], potential for hybridization with closely related species or isolated source populations [32], introduction history, and stochastic forces [33]. Indeed, the complex relationships that exist between quantitative genetic variation and differential migration-selection regimes across species ranges [34,35,36] suggest that comparative evolutionary advantage in NIPS is likely to be highly species- and population-specific.

In this paper we investigate evidence for NIPS advantage using sympatric populations of two closely related stipoid grasses from south-eastern Australia (the native *Austrostipa bigeniculata* and the exotic invasive *Nassella neesiana*) exposed to simulated climate change-type warming and drought, achieved via *in situ* manipulation of rainfall, atmospheric temperature and atmospheric CO_2_ concentrations [CO_2_]. We were interested in quantifying NIPS advantage under drought conditions because although extreme climatic events (ECEs) are known to have profound and persistent impacts on plant populations and communities [37,38,39,40,41,42], few studies have considered the ways in which they alter invasion dynamics [43,44]. Even less is known about whether the typical lack of NIPS advantage under resource stress (e.g., nutrients, light; [27]) might also apply to water availability. Given the increasing severity and duration of ECEs worldwide [45,46,47], developing a better understanding of these processes will be essential for more accurate prediction of invasive species behavior under future global change.

Here, we report the results of a study which compares the growth and reproduction of four populations of both study species collected along a 200 km temperature-aridity gradient in SE Australia under a range of current and projected (year 2050 CE, henceforth 2050; see [48]) climate regimes. In addition to testing for overall NIPS advantage in *N. neesiana*, we tested two central hypotheses derived from theory, the first (H1) relating to NIPS advantage under climate change, and the second (H2) relating to the effects of warming and CO_2_ enrichment on drought severity.

H1: NIPS advantage should increase under more favorable growing conditions (warming during winter) and decline under more stressful growing conditions (drought).

H2: Reduced growth and reproduction during drought in both species should be exacerbated by atmospheric warming but ameliorated by CO_2_ enrichment.

We also investigated whether NIPS might have enhanced evolutionary potential under future climate change by comparing levels and patterns of intra- and inter- population trait variation and heritability in both species under common garden conditions.

## 2. Methods

### 2.1. Study Species

Two C3 stipoid grasses were selected for comparison in the study: the Australian native *Austrostipa bigeniculata* (Hughes) S.W.L. Jacobs & J. Everett and the nonindigenous *Nassella neesiana* (Trin. & Rupr.) Barkworth (Chilean needle grass). Until recent taxonomic [49] and molecular data [50] supported the recognition of *Austrostipa* and *Nassella*, both species were members of the genus *Stipa*.

In Australia, *Nassella neesiana* is taxonomically difficult to distinguish from the similar species *Nassella leucotricha* (Trin. & Rupr.) R.W.Pohl (Texas needle grass). Until recently, all specimens collected from the study area, and the Australian Capital Territory (ACT), have been identified as *N. neesiana*. Recently, however, some have been re-identified as *N. leucotricha sensu* [51], primarily on the basis of length of the seed corona (>1.5 mm and <1.5 mm in *N. leucotricha* and *N. neesiana* respectively). However, in Australia and possibly in the Americas there is a lack of clarity regarding the morphological distinctiveness of these species, and taxonomic treatments contain some inconsistencies (c.f., [52,53,54]) that makes accurate identification difficult.

For example, some authors indicate that corona length overlaps in the two species (e.g., 0.7–2 mm *vs*. 0.5–1 mm; [52]). Examination of specimens from across Australia also suggests that coronal morphology exists as a continuum rather than as discrete groups, and indeed most morphological characters appear to overlap in both species [52]. Corona lengths ranged from 1.2–2.1 mm long in the four populations studied in this paper, suggesting possible affiliation with *N. leucotricha sensu* [52]; (but see [51] and [54]). Due to the poor current circumscription of the two species in Australia, and for consistency with the existing literature, in this paper were refer to all populations as *Nassella neesiana sens. lat*. However, we explicitly note that future work may identify all populations as *N. leucotricha*, or even reveal the presence of multiple taxonomic entities.

**Figure 1:**
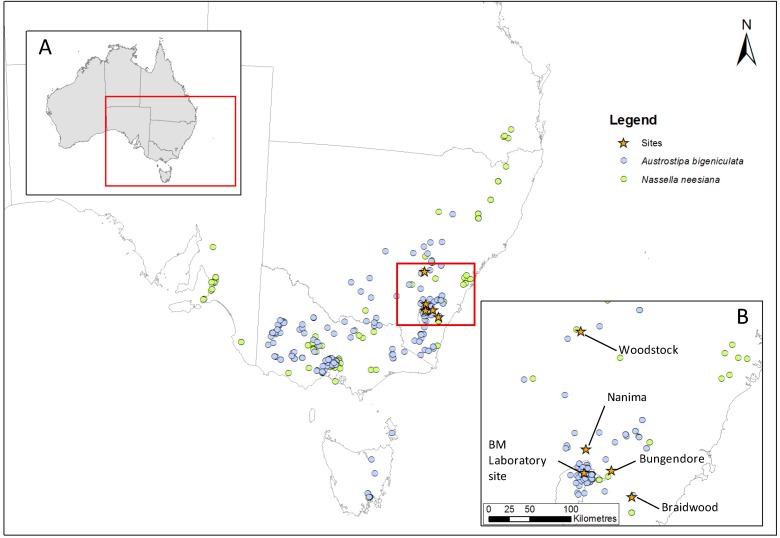
Schematic showing the study region (inset A) and herbarium specimen collection records for *Austrostipa bigeniculata* and *Nassella neesiana* across south-eastern Australia. The specific locations of the study sites along with the Black Mountain (BM) Laboratory site where glasshouse and field studies were performed are shown in inset B. Herbarium collection records were obtained from Australia’s Virtual Herbarium; see [62].

**Figure 2:**
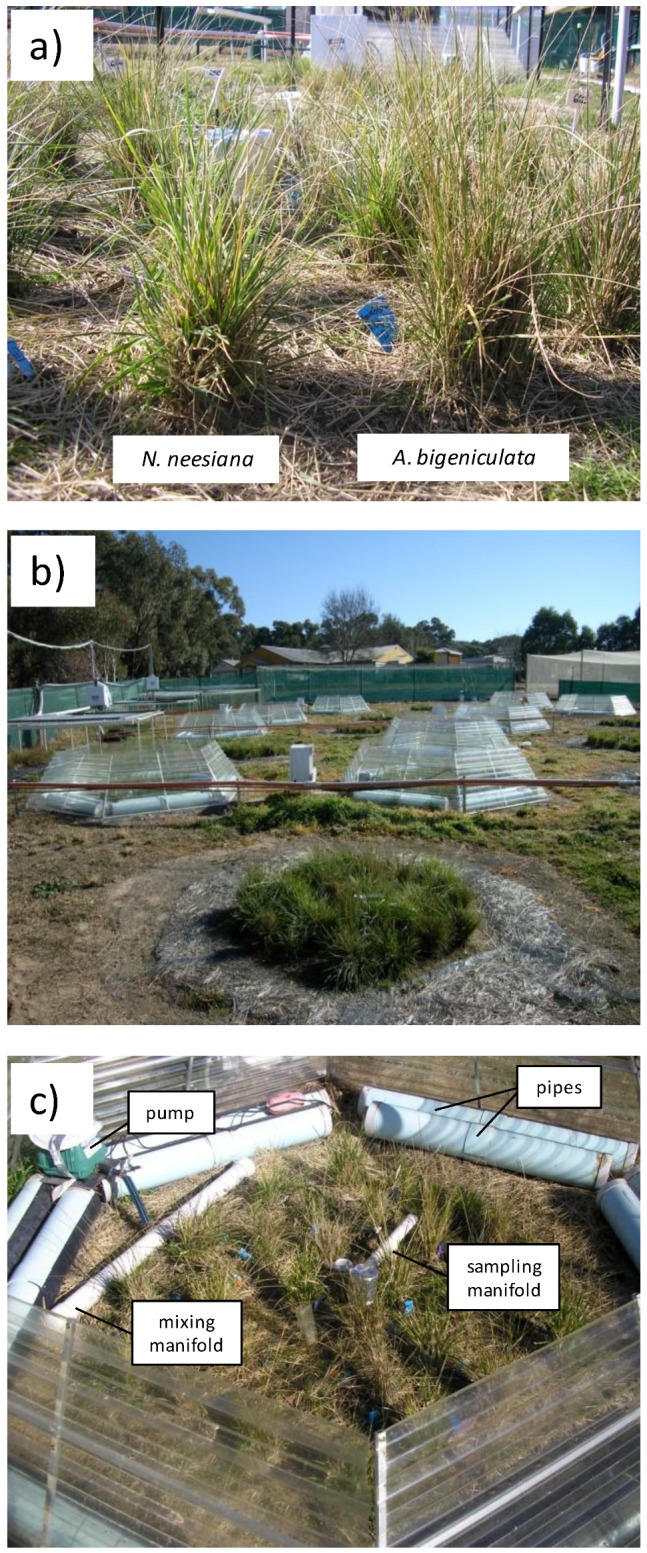
The study species and experimental study site. (**a**) Ramets of *Nassella neesiana* and *Austrostipa bigeniculata* growing in a study plot. (**b**) The experimental site at Black Mountain, Australian Capital Territory (ACT), Australia, showing open-top chambers. The view is to the west. (**c**) Close-up of the inside of a chamber, showing pipes (containing water) arranged around the outside of the plot, along with the CO_2_ injection pump, mixing manifold and sampling manifold.

*Austrostipa bigeniculata* is a common to dominant species which grows widely across SE Australia, both in natural and derived grasslands [55,56,57]. *Nassella neesiana* was first recorded in Australia in 1934 and is now one of the nation’s worst weeds [54,58]. Infestations occur across SE Australia, especially in areas which receive more than 500 mm annual rainfall (Figure 1). In southern New South Wales (NSW), *A. bigeniculata* and *N. neesiana* have strongly overlapping ranges. Both species form tall (up to 1 m excluding flowering stems) perennial tussocks (Figure 2a). In contrast to *A. bigeniculata*, which mainly produce panicle seeds, *N. neesiana* also frequently produces cleiostogenes or “stem seeds” (chasmogamous seeds) at the nodes and bases of the stems [59], a feature that in other stipoid grasses increases fitness under stressful growing conditions, including heavy grazing and fire [59,60], soil moisture deficiencies [61] and other climatic cues.

### 2.2. Site Selection and Description

Populations of *A. bigeniculata* and *N. neesiana* were collected from four sites along a 200 km transect in SE Australia (Figure 1): Braidwood (S 35.47°, E 149.79°), Bungendore (S 35.21°, E 149.48°), Nanima (S 35.01°, E 149.10°) and Woodstock (S 33.75°, E 148.84°). Temperature and potential evaporation (Table 1), and hence aridity and drought severity, increase with distance west along the transect, and vegetation changes considerably. We attempted to spread collection sites evenly along the transect, but were limited by the availability of *N. neesiana*, and so the two central sites (Bungendore and Nanima) had similar climatic characteristics (Table 1). The Braidwood site lay near the eastern range margin of both species, but the Woodstock site lay at the western margin of only *N. neesiana*—a few scattered populations of *A. bigeniculata* exist further to the west (Figure 1).

### 2.3. Specimen Collection and Propagation

Up to 50 *A. bigeniculata* and *N. neesiana* tussocks were collected from within a 1 ha collection area at each of the four study sites (Figure 1) between August and early December 2010. Randomly selected tussocks (at least 10 cm in diameter and 1 m apart) were removed from the ground and transported to the CSIRO Black Mountain Laboratory site (S 35.27°, E 149.11°; Figure 1) in moist sacks. Tussocks were then planted in 20 cm diameter pots in a 25 °C/15 °C day/night glasshouse and watered regularly for two weeks until they produced new leaf material. Pots contained standard high fertility potting mix and received one application of Scotts^®^ Osmocote^®^ Exact^®^ 3–4 month programmed release fertilizer (16% N as nitric and ammoniacal nitrogen, 9% P_2_O_5_, 12% K_2_O, 2% MgO) at the time of planting to ensure adequate nutrient availability. Watering was performed as required. Plants established during late winter (August) were slower to respond than later collections and so tussocks tended to quickly converge in size.

**Table 1:** Climate data (1910–2009) for the four collection sites. Data were obtained from the SILO enhanced climatic data bank hosted by the Queensland Climate Change Centre of Excellence [63]. Tmax = maximum temperature; Tmin = minimum temperature; PET = potential evapotranspiration.

		Collection site			
		Braidwood	Bungendore	Nanima	Woodstock
*Average Tmax* (°C)					
	Summer	24.9	26.9	27.4	30.0
	Autumn	19.1	19.7	20.1	22.3
	Winter	12.4	11.9	12.0	13.7
	Spring	18.8	19.4	19.7	22.0
	Annual	18.8	19.5	19.8	22.0
*Average Tmin (°C)*					
	Summer	11.9	12.6	12.8	14.4
	Autumn	7.0	7.1	7.3	8.8
	Winter	1.3	1.1	1.4	2.9
	Spring	6.3	6.4	6.5	7.7
	Annual	6.6	6.8	7.0	8.5
*Total precipitation (mm)*					
	Summer	208.7	166.8	152.6	177.2
	Autumn	192.4	151.5	147.2	140.5
	Winter	159.0	156.9	175.0	177.0
	Spring	186.8	184.0	183.8	182.8
	Annual	746.9	659.2	658.6	677.5
*Total PET (mm)*					
	Summer	409.0	462.0	467.9	518.4
	Autumn	214.0	238.4	238.5	269.7
	Winter	120.2	124.4	121.9	132.0
	Spring	298.5	317.9	315.9	346.4
	Annual	1,041.7	1,142.7	1,144.2	1,266.5

Once established, 24 healthy tussocks from each of eight populations (2 species × 4 collection sites) were selected, removed from pots, broken into four to seven equally sized ramets, and established in new 10 cm pots. All ramets taken from a single plant are henceforth referred to as a clone line, with each set of 24 clone lines representing a population. Ramets were allowed two weeks in the glasshouse to establish and were then moved outdoors for a further two weeks. Four well-established and (as much as possible) similarly-sized ramets were then selected from each clone line for use in the subsequent field trial. A total of 768 plants (4 ramets per clone line × 24 clone lines per population × 8 populations) were used in the trial.

### 2.4. Field Trial Design and Establishment

**Figure 3:**
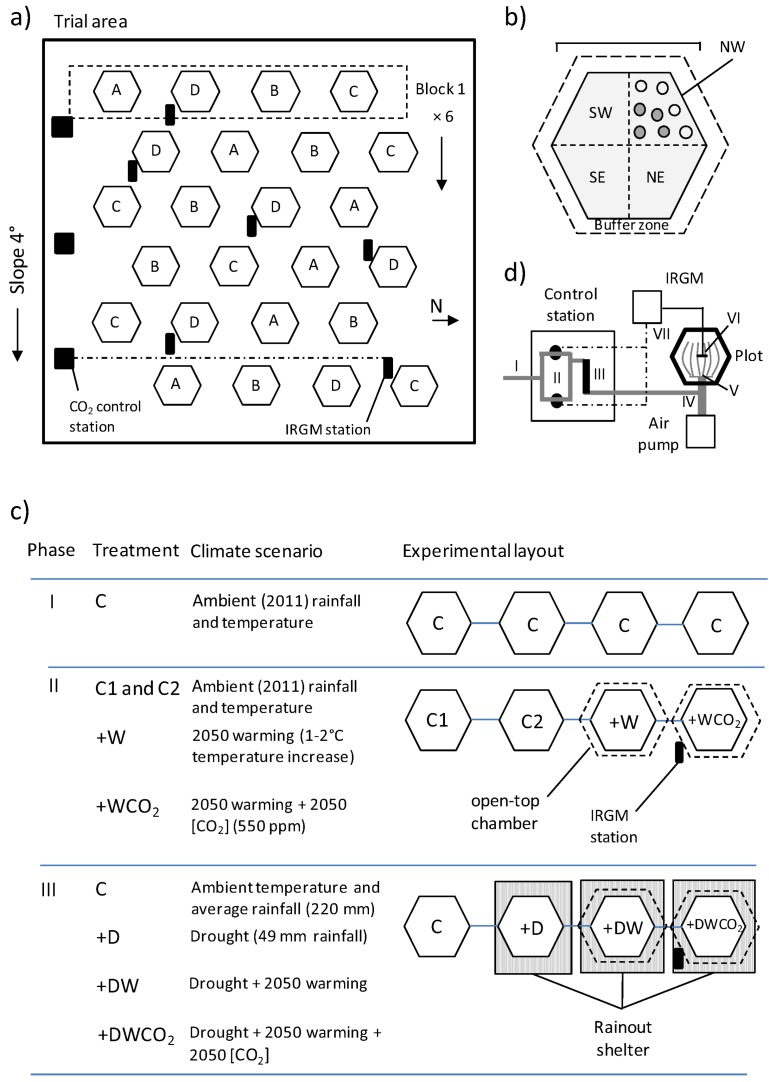
Design and layout of the field experiment. (**a**) Layout of the study site. Each block (n = 6 total) contained four plots, each of which was assigned to one of four climate treatments in Phase III of the experiment (C, +D, +DW and +DWCO_2_; labeled A–D) as defined in (c). (**b**) Layout of a study plot (n = 24 total). Hexagon-shaped plots (2.2 m^2^) were divided into four equal-sized quadrants (NW, NE, SE and SW), each of which contained eight plants—four inside (grey circles) and four edge (white circles). (**c**) Treatment structure, experimental layout and simulated climate scenarios during Phases I, II and III of the experiment. Note that the four climate treatments were randomly allocated to plots within each block. (**d**) Basic design of the CO_2_ enrichment system. A line of pure CO_2_ (I) is supplied to each CO_2_-enriched chamber via a CO_2_ control station [shown in (a)], where it is split into two lines, the flow of each being controlled by a solenoid valve (II). Both CO_2_ lines then merge, pass through a flow meter (III), and enter a main air line (IV) which is supplied by a 60 L min^−1^ air pump. The air and pure CO_2_ then mix in a mixing manifold (V) and enter the plot via a series of porous CO_2_ injection tubes. The CO_2_ concentration is sampled by an infra-red gas monitor (IRGM) via a sampling manifold placed in the center of the plot (VI); the IRGM controls the solenoids via relays (VII) with different set-points for each of the solenoids (here 550 ppm and 575 ppm).

The field trial consisted of twenty-four 2.2 m^2^ hexagon-shaped plots located within a 0.25 ha trial area at the CSIRO Black Mountain Laboratory site (Figure 3a). Plots were arranged into six blocks of four plots each, with blocks perpendicular to the aspect of the trial site (sloping 4° to the east; Figure 3a). This blocking arrangement captured potential site-level variation in soil water, soil nutrient level and shading associated with topographic position. The trial site was surrounded by a 2 m tall rabbit-proof fence covered with fine plastic mesh which reduced air flow and improved economy and control of CO_2_ enrichment. Because of this design, and the proximity of buildings and trees to the site, we explicitly limited experimental inference to the low wind speeds (0–3 ms^−1^ at 300 mm above ground) that occurred across the site during the study period.

The experiment was conducted in three discrete phases (Figure 3c), the first a common garden experiment, and the second and third involving climatic manipulation. Plots within blocks were allocated to one of four Phase III treatments (described below; labeled for convenience A–D in Figure 3a) corresponding to the climatic scenarios listed in Figure 3c. Each of the four treatments was replicated six times, once in each block (Figure 3a). The specific placement of ramets within plots ensured correct experimental design during all phases of the experiment.

Ramets were allocated to blocks and plots using a stratified random experimental structure in which one ramet from each of the 24 clone lines of each population occurred once in each Phase III treatment (Figure 3a,c; see below). Ramets from a given clone line (*n* = 4) were randomly allocated to different climate treatments (A–D) and then randomly to different blocks, thus reducing correlation between block and clone line. To balance the design, each plot contained four plants (one ramet from each of four randomly selected clone lines) from each population, for a total of 32 experimental plants. Within each plot, the 32 plants were arranged in a stratified random manner to reduce any effect of plot location on growth and reproduction. Plots were divided into four quadrants of equal size (NW, NE, SE and SW), each of which contained eight plants—a single ramet from each of eight populations (Figure 3b). Each plant was then randomly allocated to one of eight positions within the quadrant: half being along the edge of the quadrant and half on the inside (Figure 3b). This design was maximally efficient given the objectives and structure of the experiment and ensured that any positional and neighbor effects were randomly distributed with respect to species, population, and clone line.

Ramets were planted into plots during December and January 2011 and loosely packed with soil up to the base of the tussock crowns. Plants were watered until established (producing new green leaf material) and dead individuals were replaced with genetically identical ramets within 2–3 weeks. Soil in the plots consisted of a medium to high fertility sandy loam (Colwell P > 50 mg kg^−1^; total N > 0.1%; total C = 1.2%–1.9%; and adequate K, Mg and Na), low salinity (electrical conductivity = 40–72 μS cm^−1^), and low to moderate acidity (calcium chloride [CaCl_2_] pH = 4.9–6.3). Sulphur levels were at or just below levels associated with adequate pasture availability (2.5–8.6 mg kg^−1^).

### 2.5. Field Trial Treatments and Climate Scenarios

The experiment proceeded in three phases (Figure 3c), each designed to test a different set of questions relevant to plant adaptation under climate change. During Phase I (February to April 2011) all plants were grown in a common garden experiment under ambient climatic conditions. This allowed determination of population-level quantitative variation and broad sense heritability [64,65] of growth variables without confounding by climate treatment effects (Figure 3c). During this period, total precipitation (161 mm) was just above the long term (1910–2009) Canberra average (145 mm). Observed mean maximum and minimum ambient screen temperatures were close to the 1980–1999 average (23.1 °C *vs*. 24.1 °C and 10.7 °C *vs*. 10.2 °C respectively). This timeframe is used for comparison with projected climate regimes (see [48]).

In Phase II (late April to June 2011) large open-top chambers (see [66]) were placed over two of the four plots in each block, one chamber of which received CO_2_ enrichment to 550 parts per million by volume (ppm). Chambers raised atmospheric temperatures by 1–2 °C, thus simulating approximate year 2050 warming [48], while 550 ppm [CO_2_] was adopted as a plausible scenario for 2050. Each block therefore contained two control plots with an ambient (2011) climate regime (C1 and C2) and a single plot containing 2050 warming (+W treatment) and 2050 warming + [CO_2_] (+WCO_2_). Specific treatment details are provided in Figure 3c. The objective of Phase II was to quantify the effects of future atmospheric warming and CO_2_ enrichment on plant populations under non-drought conditions during winter, when water availability is usually high. During this phase rainfall was below average (22 mm *vs*. 89 mm) but plants did not visibly show moisture stress. Maximum ambient temperatures were near average (14.6 °C *vs*. 14.2 °C) but night minima were below normal (0.6 °C *vs*. 2.6 °C).

During Phase III (July to early November 2011) we imposed drought on +W and +WCO_2_ treatments and one of the control replicates (C2), resulting in four new treatments: (1) control (C), (2) drought (+D), (3) drought + warming (+DW), and (4) drought +warming + elevated [CO_2_] (+DWCO_2_; Figure 3c). Control plots received 221 mm of rain during this period, equal to the Canberra average for the period, while drought-affected plots received 49 mm, only 22% of average. Drought rainfall was below the minimum (and first percentile) July to November 10 rainfall observed at Canberra and the four collection sites in the last century (65–85 mm), and so was clearly extreme. We note, however, that plot soil profiles also contained around 80 mm of plant available water in early July 2011, based on an estimated minimum plant available volumetric soil water content of 8% and a 70 cm root depth. Maximum ambient screen temperatures were above the 1980–1999 average (17.4 °C *vs*. 15.4 °C) but minimum temperatures were average (2.9 °C *vs*. 3.2 °C).

Atmospheric CO_2_ enrichment was achieved using the simple, low-cost injection system previously reported in [66], but with a few modifications for larger-scale field experimentation (Figure 3d). CO_2_ control is most precise during the day when photosynthetically active radiation (PAR) is highest but poorer on still, cold winter mornings and evenings when [CO_2_] tends to exceed the target range of 550 ± 110 ppm. To conserve CO_2_ we turned off the injection system outside of daylight hours and when wind speeds exceeded 10 ms^−1^ (approximately 6% of total daylight hours). Drought was imposed on plots by covering them with rainout shelters (clear plastic tents) immediately prior to rainfall. Shelters were removed as soon as possible to reduce PAR interception (roughly 10%–20% of PAR during use) and were only in place for approximately 17% of total daylight hours.

### 2.6. Data Collection

To begin Phase I in February 2011 we cut all plants back to a height of 150 mm. This standardized plant size and allowed for accurate quantification of growth parameters but did not visibly reduce plant vigour. After 14 days we recorded leaf elongation, expressed as a daily rate (LER; mm day^−1^), and the height to width ratio (HWR) of all tussocks, a measure of growth form in which higher score indicates a more upright stature. Width was determined by averaging two perpendicular measurements made across the tussock. In mid April 2011 we harvested all biomass above 150 mm, and determined the daily biomass accumulation rate (BAR; mg day^−1^). We also determined the width, height (excluding stems), and the mid and basal diameters (compressed gently by hand) of all tussocks, and determined tussock volume (cm^3^). We also recorded the rate of flowering stem production (StemPR; stems plant^−1^).

During Phase II, LER and BAR were again determined using the same methods as above; the amount of clipped material was small due to slow winter tussock growth and so impacts on plant growth were minimal. Phase III commenced in July 2011 with the watering of all plots to common soil water content (SWC) which allowed for the subsequent quantification of treatment effects on soil water. The drought regime was then imposed on C2, +W and +WCO_2_ treatments, resulting in C, +D, +DW and +DWCO_2_ treatments (Figure 3c). In August 2011 we again determined LER based on clipping a small section of tussock. We terminated the experiment on November 10 when some drought-stressed plants were approaching death and then commenced harvesting above-ground biomass (AGB) from tussocks. Due to time constraints, we only subsampled 24 ramets per population (25% of the total), evenly distributed across treatments. To ensure that no plants were killed by the procedure, we removed only a proportion of the basal area of each tussock. AGB was estimated for each plant by dividing the harvest weight (dried for 3 days at 70 °C) by the proportion of tussock basal area sampled of the total, assuming tussock symmetry. We also determined maximum length of flowering stems (MaxSL; cm), the flowering stem production rate (stems dm^−2^ of tussock basal area), and the rate of floret production per inflorescence by counting floret numbers on up to ten inflorescences (if available) on each plant. Finally, we determined the total basal expansion (BasalE; mm) of each experimental plant over Phases II and III by comparing basal diameter in April and the final harvest date.

Plot temperatures were recorded in the center of each plot at 10 cm above the soil surface using Hobo^®^ Pendant UA-002-64 data loggers. To eliminate direct solar exposure, loggers were placed in small screens consisting of upturned pots covered in reflective silver tape. Data were recorded in two plots per treatment for 2–4 weeks during each month except in June when some loggers failed. SWC data were collected from the top 50–70 cm of the soil profile using neutron access tubes and a CPN Corporation Hydroprobe^®^ (Martinez, Ca). Volumetric SWC was calculated using the calibration equation:

SWC (% vol) = 2.0 × 10^−7^*x*^2^ − 1.5 × 10^−4^*x* + 1.82

where *x* = the neutron probe reading, which was determined by comparing the SWC of soil core sections with probe readings taken at the time of removal. For a more detailed description of the methodology see [40].

### 2.7. Data Analyses

Growth and reproduction data were analyzed using general linear mixed model (LMM) analysis with model structure dependent on the experimental phase. Phase I models contained species (*A. bigeniculata vs*. *N. neesiana*), collection site (Braidwood, Bungendore, Nanima and Woodstock), the species × site two-way interaction term, planting (original *vs*. replacement), plot quadrant (Quad: NW, NE, SW and SE), quadrant position (Qpos: inside *vs*. edge) and Quad × Qpos, Quad × species, Qpos × species and Quad × Qpos × species interaction terms as fixed predictors (Table 2). Block (*n* = 6), plot within block (*n* = 4) and clone line within population (*n* = 24) were included as random predictor variables. Since data were missing for some variables block, Quad × Qpos × species and planting terms were removed from some models to ensure model convergence. Models and model degrees of freedom were estimated using REML and Satterthwaite methods respectively. We also determined estimated clone line means for each population and site and tested for differences among means using Tukey-Kramer—adjusted post-hoc means tests. Data were transformed where required to improve data conformity with model assumptions.

For one variable, April flowering stem production, data were highly skewed with many ramets producing no stems. Here, no transformation was entirely appropriate and so the data were analysed using generalized linear model analysis with stem count modeled as a Poisson distribution with log link and species, site, species × site, planting, Quad, and Qpos as predictor variables. Data were analysed using SAS Proc Mixed and Proc Genmod version 9.1 (SAS Institute Inc., Cary, NC, USA). Broad sense heritability (*H*^2^) of clonal lines was estimated for LER and BAR as *H*^2^ = V_G_/V_P_ where V_G_ is the genetic variance among clone lines and V_P_ is the total phenotypic variance [65,67]. Broad sense heritability represents an upper limit estimate of narrow sense heritability, estimates of which are based only on additive genetic variance. Variance estimates were obtained using SAS Proc Varcomp version 9.1 (SAS Institute Inc., Cary, NC, USA).

Linear mixed models for Phase II variables and LER in August contained the same terms as above but also climate treatment (Treat) (*i.e.*, C1, C2, +W and +WCO_2_ or C, +D, +DW and +DWCO_2_) and associated two- and three-way interaction terms between Treat, species and site. We also included two-, three- and four-way interaction terms between Treat, Quad, Qpos and species, but to reduce model complexity we excluded higher order interaction terms between Quad, Qpos, species and site. As before, block, plot within block and clone line within population terms were included as random factors. Except for LER in August, Phase III data (Table 3) were collected by sampling a restricted set of plants and so were analysed using more simple models which excluded higher-order interactions involving the spatial terms Quad and Qpos (except the Treat × Qpos term), all of which were non-significant in Phase II models. Block and plot within block terms were included as random variables but clone line within population was excluded. We again compared predicted treatment × species (interaction) means for each variable, and where significant, species × site means, using Tukey-Kramer—adjusted post-hoc means tests. Data were analysed using SAS Proc Mixed version 9.1 (SAS Institute Inc., Cary, NC, USA).

**Table 2:** Results of linear mixed model analysis of trait data collected during Phase I of the experiment. Data in columns adjacent to fixed variables are F and associated *p* values (*** = *p* < 0.001; ** = *p* < 0.01; * = *p* < 0.05, ns = *p* ≥ 0.05); for random variables Z and *p* are provided. Estimated population means sharing the same letter did not differ at the 0.05 significance level.

					Dependent variable											
					Leaf elongation rate (LER)		Height to width ratio (HWR)		Tussock width (Wid)		Tussock volume (Vol)		Height to width ratio (HWR)		Biomass accumulation rate (BAR)	
Experimental phase measured					I		I		I		I		I		I	
Month					February		February		February		April		April		Feb-April	
Units					mm day^−1^		none		mm		cm^3^		none		mg day^−1^	
Transformation					sqrt(×)		ln(×)		sqrt(×)		sqrt(×)		ln(×)		sqrt(×)	
*Model fixed effects (*F*, p)*																
	Species				52.2	***	831.2	***	540.3	***	399.9	***	719.1	***	18.5	***
	Site				129.8	***	82.1	***	49.7	***	35.7	***	31.8	***	57.0	***
	Species × Site				115.2	***	30.3	***	6.3	***	3.9	*	4.4	**	28.0	***
	Planting				68.5	***	2.8	ns	58.0	***	75.1	***	-		103.2	***
	Quadrant (Quad)				5.4	**	2.8	*	2.3	ns	0.1	ns	1.4	ns	5.6	***
	Quadrant Position (Qpos)				1.4	ns	25.1	***	18.1	***	50.4	***	78.5	***	1.1	ns
	Quad × Qpos				1.3	ns	3.1	*	1.5	ns	1.6	ns	3.5	*	0.8	ns
	Quad × Species				1.1	ns	1.4	ns	2.0	ns	2.8	*	0.9	ns	3.6	*
	Species × Qpos				2.0	ns	0.5	ns	0.0	ns	2.7	ns	2.1	ns	0.1	ns
	Quad × Species × Qpos				0.3	ns	0.7	ns	0.8	ns	0.5	ns	-		0.1	ns
*Random effect (*Z*,* *p)*																
	Clone line				2.8	**	3.7	***	2.1	*	3.0	**	0.1	ns	4.7	***
	Block				1.4	ns	0.4	ns	1.2	ns	-		0.8	ns	1.2	ns
	Plot (Block)				1.2	ns	1.5	ns	1.6	ns	2.1	*	0.2	ns	2.4	**
*Estimated population means*																
	*A. bigeniculata*	Braidwood			2.94	cd	0.00	cd	17.3	bc	17.6	c	−0.12	c	13.8	bc
		Bungendore			3.08	bc	0.26	a	15.8	d	13.9	d	0.07	a	12.5	c
		Nanima			3.14	b	0.07	bc	17.2	c	18.2	c	−0.12	bc	16.4	b
		Woodstock			3.13	bc	0.15	ab	16.8	c	16.0	cd	−0.03	b	16.1	b
	*N. neesiana*	Braidwood			2.76	d	−0.50	f	20.4	a	27.8	a	−0.48	e	15.9	b
		Bungendore			2.40	e	−0.32	e	18.0	b	21.9	b	−0.39	d	12.0	c
		Nanima			2.38	e	−0.61	f	19.7	a	28.4	a	−0.53	e	13.8	bc
		Woodstock			3.86	a	−0.07	d	20.4	a	22.5	b	−0.35	d	24.6	a

**Table 3:** Results of linear mixed model analysis of trait data collected during Phases II and III of the experiment. Climate treatment acronyms are provided in Figure 3. Columns adjacent to fixed variables contain F and associated *p* values (*** = *p* < 0.001; ** = *p* < 0.01; * = *p* < 0.05, ns = *p* ≥ 0.05); for random variables Z and *p* are provided. Estimated means sharing the same letter did not differ at the 0.05 significance level. Only a restricted set of interactions involving quadrant and quadrant position are included; all others were not significant at the 0.05 significance level. Full model details are provided in the text. For species, *Aubi* = *Austrostipa bigeniculata* and *Nane* = *Nassella neesiana*.

					Dependent Variable																				
					Leaf elongation rate (LER)				Biomass accumulation rate (BAR)						Leaf elongation rate (LER)					Basal diameter (Basal D)						
Experimental phase measured					II				II						III					III						
Month					June				June						August					November						
Units					mm day^−1^				mg day^−1^						mm day^−1^					mm						
Transformation					none				ln(×)						none					none						
*Model fixed effects* (F,*p*)																									
	Species				60.7		***		65.1		***				166.1		***			155.5		***			
	Site				44.2		***		23.1		***				15.4		***			10.8		***			
	Species × Site				31.2		***		10.5		***				36.8		***			0.7		ns			
	Climate treatment (Treat)				3.2		ns		2.4		ns				10.6		***			1.3		ns			
	Treat × Species				6.5		***		5.2		**				2.9		*			1.3		ns			
	Treat × Site				1.8		ns		0.6		ns				1.9		ns			0.9		ns			
	Treat × Species x Site				0.9		ns		0.6		ns				0.9		ns			0.5		ns			
	Planting				31.3		***		99.4		***				37.7		***			2.2		ns			
	Quadrant (Quad)				1.4		ns		0.9		ns				1.8		ns			1.0		ns			
	Quadrant position (Qpos)				0.3		ns		1.0		ns				15.9		***			24.9		***			
	Treat × Quad				2.0		*		0.8		ns				0.3		ns			-					
	Treat × Qpos				4.0		**		3.5		*				4.0		*			0.5		ns			
*Random effects (*Z*,* *p)*																									
	Clone line				1.6		ns		2.7		**				3.8		***			-					
	Block				1.4		ns		1.3		ns				1.3		ns			0.1		ns			
	Plot(Block)				2.3		*		2.2		*				2.1		*			1.0		ns			
*Estimated species x treatment means*																									
	*Phase II*	*Phase III*	Species																						
	C1	C	*Aubi*		2.48		bc		2.73		b				5.87		b			57.1		b			
	C2	+D	*Aubi*		2.56		b		2.74		b				6.15		b			47.2		b			
	+W	+DW	*Aubi*		3.05		a		3.20		ab				7.33		a			59.4		b			
	+WCO_2_	+DWCO_2_	*Aubi*		3.05		a		3.10		ab				7.25		a			55.5		b			
	C1	C	*Nane*		2.37		bc		3.31		a				4.67		c			84.1		a			
	C2	+D	*Nane*		2.27		c		3.27		a				4.84		c			84.3		a			
	+W	+DW	*Nane*		2.51		bc		3.42		a				5.49		b			86.5		a			
	+WCO_2_	+DWCO_2_	*Nane*		2.48		bc		3.37		a				5.62		b			92.3		a			
				**Dependent Variable**																					
				**Basal expansion (BasalE)**				**Maximum stem length (MaxSL)**				**Above ground biomass (AGB)**				**Stem Prod Rate (StemPR)**					**Floret production rate (FlorPR)**				
Experimental phase measured				III				III				III				III					III				
Month				November				November				November				November					November				
Units				mm				cm				g				stems dm^−2^					florets infl^−1^				
Transformation				none				none				sqrt(×)				sqrt(×)					none				
*Model fixed effects* (F,*P*)																									
	Species			11.8		***		0.2		ns		35.2	***			39.0		***			53.6		***		
	Site			2.5		ns		2.1		ns		7.3	***			10.3		***			0.9		ns		
	Species × Site			1.4		ns		1.8		ns		2.8	*			3.1		*			2.0		ns		
	Climate treatment (Treat)			1.1		ns		1.9		ns		0.6	ns			1.0		ns			2.9		ns		
	Treat × Species			0.6		ns		3.9		*		1.3	ns			4.8		**			3.0		*		
	Treat × Site			0.9		ns		0.1		ns		0.6	ns			1.6		ns			0.8		ns		
	Treat × Species x Site			0.5		ns		0.3		ns		0.5	ns			0.8		ns			0.8		ns		
	Planting			1.6		ns		0.3		ns		2.4	ns			0.0		ns			1.0		ns		
	Quadrant (Quad)			0.1		ns		0.8		ns		0.3	ns			0.5		ns			0.3		ns		
	Quadrant position (Qpos)			2.0		ns		6.5		*		27.2	***			0.4		ns			12.3		***		
	Treat × Quad			-				-				-				-					-				
	Treat × Qpos			1.3		ns		1.3		ns		0.0	ns			0.8		ns			0.8		ns		
*Random effects (Z,* P*)*																									
	Clone line			-				-				-				-					-				
	Block			0.6		ns		0.2		ns		0.3	ns			0.5		ns			-				
	Plot(Block)			0.6		ns		1.8		*		1.3	ns			0.8		ns			0.8		ns		
*Estimated species x treatment means*																									
	*Phase II*	*Phase III*	Species																						
	C1	C	*Aubi*	4.1		ab		137.4		a		7.6	bc			1.66		b			64.4		a		
	C2	+D	*Aubi*	1.9		ab		125.2		a		7.4	bc			1.85		a			61.6		a		
	+W	+DW	*Aubi*	1.8		b		105.6		a		7.4	bc			1.50		bcd			43.3		bc		
	+WCO_2_	+DWCO_2_	*Aubi*	6.3		ab		110.1		a		7.0	c			1.61		bc			53.0		ab		
	C1	C	*Nane*	7.6		ab		128.1		a		9.9	a			1.45		bcd			38.8		bc		
	C2	+D	*Nane*	11.2		ab		112.5		a		8.3	abc			1.23		d			35.8		c		
	+W	+DW	*Nane*	12.4		a		116.3		a		9.7	ab			1.37		bcd			36.3		bc		
	+WCO_2_	+DWCO_2_	*Nane*	18.7		a		129.0		a		9.8	ab			1.32		cd			36.0		c		

We also tested for differences in mean volumetric soil water content (SWC%) and change in soil water content (∆SWC) across a range of time intervals during the Phase III of the experiment. We determined SWC for all plots at depths of 10 cm, 20 to 40 cm and 50–70 cm on 7 July, 1 August, 23 August, 6 October and 10 November 2011, and determined ∆SWC for the intervals 7 July to 1 August, 23 August to 6 October, and 6 October to 10 November. Variables were analyzed using linear mixed model analysis with climate treatment and block as fixed predictor variables; predicted treatment means were tested using Tukey-Kramer—adjusted post-hoc means tests. All LMM analyses were performed using SAS Proc Mixed version 9.1.

## 3. Results

### 3.1. Temperature and [CO_2_]

Warming generated by the open-top chambers was broadly in line with previous observations [66]. Daytime (06:00–17:30) warming was generally around 1–2 °C in all months except July when it ranged from 0–1 °C (Table 4). Daily maximum temperatures were generally elevated more than mean daily temperatures. At night (18:00–05:30) temperatures were usually 0.5–1.5 °C warmer inside chambers although extreme minima were as much as 2.2 °C higher (Table 4). Daytime mid-canopy atmospheric [CO_2_] averaged 576 ppm across the six enriched chambers (range 541–612 ppm).

**Table 4:** Temperatures observed under different climate treatment regimes during the experiment. Actual temperatures are shown for ambient control plots (shaded) while deviations from the control are shown for treatments. Treatment acronyms are described in Figure 3. Tav = average temperature, Tmax = average daytime maximum, Temax = extreme (highest) daytime maximum, Tmin = average night minimum, Temin = extreme (lowest) night minimum.

	Climate Treatment		Day				Night		
Month			Tav	Tmax	Temax		Tav	Tmin	Temin
May	Control		9.9	15.5	20.7		3.0	0.3	-3.1
	+W		+0.6	+1.6	+2.0		+0.9	+1.1	+1.1
	+WCO_2_		+0.5	+1.0	+1.1		+1.1	+1.3	+1.3
July	Control		8.3	13.6	19.0		3.9	0.9	−2.5
	+D		0.0	0.0	-0.4		+0.1	+0.1	−0.2
	+DW		+0.5	+1.0	-0.1		+0.4	+0.5	+0.2
	+DWCO_2_		+0.4	+0.6	-0.2		+0.4	+0.6	+0.8
August	Control		11.9	18.5	23.9		6.2	2.9	−0.9
	+D		+0.5	+0.9	+0.7		0.0	+0.1	+0.2
	+DW		+1.0	+2.0	+2.4		+0.6	+1.0	+1.6
	+DWCO_2_		+1.1	+1.5	+2.2		+0.8	+1.3	+2.0
September	Control		15.3	21.9	29.0		7.9	4.1	−1.1
	+D		+0.9	+1.4	+1.6		+0.3	+0.4	+0.6
	+DW		+1.9	+3.1	+3.1		+0.8	+1.0	+1.1
	+DWCO_2_		+1.5	+1.7	+1.0		+1.2	+1.6	+2.2
October	Control		20.7	29.2	37.1		11.7	8.6	1.8
	+D		+0.8	+0.8	+0.7		+0.3	0.0	−0.3
	+DW		+1.1	+1.9	+2.3		+0.7	+0.4	+0.4
	+DWCO_2_		+1.1	+0.6	0.0		+0.8	+0.5	+0.8

### 3.2. Soil Water Content

**Figure 4:**
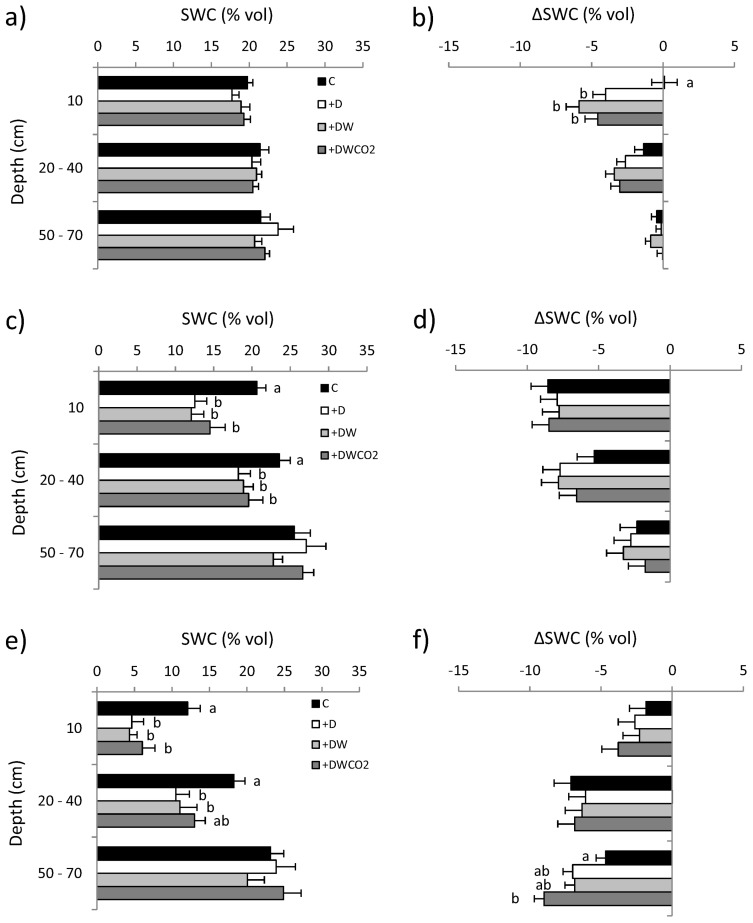
Volumetric soil water content (SWC, %) in experimental plots during winter and spring 2011. (**a**) SWC on 7July 2011 in the upper (10 cm), mid (20–40 cm) and lower (50–70 cm) soil profile. (**b**) Change in SWC over the period 7July to 1August 2011. (**c**) SWC on 23August 2011. (**d**) Change in SWC over the period 23August to 6October, 2011. (**e**) SWC on 6October 2011. (**f**) SWC change over the period 6October to 10November 2011. Data are estimated means based on six experimental plots ± one standard error. Within each depth and timeframe, means sharing the same letter are not significantly different at the *p* = 0.05 level; only those in which at least two means differed are shown.

On 7 July 2011, at the start of Phase III of the experiment, no differences in SWC (*p* > 0.2) were evident anywhere in the soil profile (Figure 4a). Plant-available water reserves were adequate (Figure 4a) and plants showed no signs of moisture stress. After 25 days (7 July–1 August) SWC change (∆SWC) in the upper profile varied significantly across treatment regimes (F_3,15_ = 8.03, *p* < 0.01), with SWC declining by close to 5% in all treatments except the control (Figure 4b). No significant differences between +D, +DW and +DWCO_2_ treatments were observed. Soil water also declined in the mid profile (Figure 4b), but treatment-level differences were not significant (F_3,15_ = 1.68, *p* = 0.21).

By August 23 significant (*p* < 0.05) differences had emerged across treatments in the upper and mid soil profile, with SWC being up to 8% lower in drought-affected plots than in control plots (Figure 4c). Differences were more pronounced in the upper profile, and again, there was no evidence that warming or atmospheric CO_2_ enrichment affected SWC (cf., +D *vs*. +DW and +DW *vs*. +DWCO_2_) (Figure 4c). Between 23 August and 6 October, SWC declined greatly throughout the soil profile (Figure 4d), leading to extreme upper profile soil water deficiencies in all treatments except the control (Figure 4e). By this time, stress (e.g., leaf rolling) was observable in plants across +D, +DW and +DWCO_2_ treatments. Over the next 34 days SWC declined rapidly in the mid and lower soil profile, but remained above 10% in control plots. Water loss was again unrelated to treatment except in the lower rhizosphere (F_3,15_ = 6.87, *p* < 0.01) where loss was greatest in the +DWCO_2_ treatment and least in the control (Figure 4f). By the end of the experiment most plants in non-control plots showed signs of either moderate or severe moisture stress.

### 3.3. Plant Growth and Reproduction

**Figure 5:**
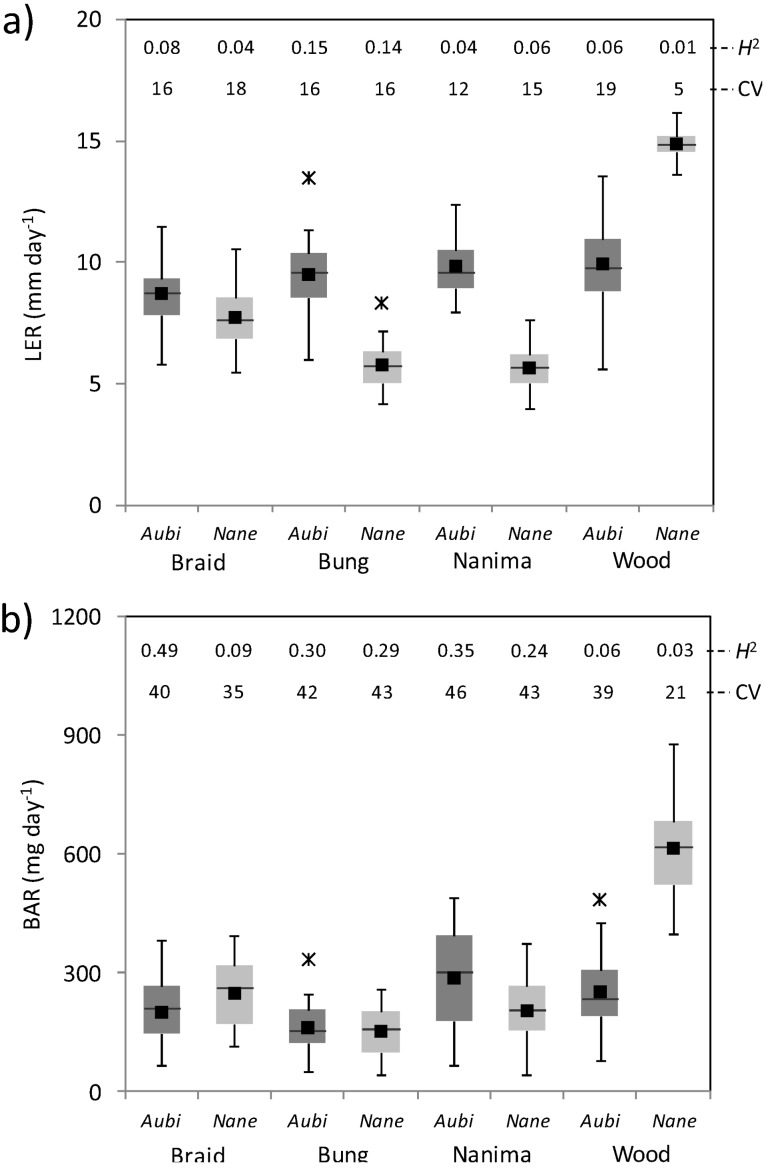
Boxplots showing growth of *A. bigeniculata* and *N. neesiana* tussocks during Phase I of the experiment. (**a**) Leaf elongation rate (LER) in February 2011. (**b**) Biomass accumulation rate (BAR) during February–April 2011. Data are based on estimated means for each clone line (n = 24) within each of the eight plant populations (four *A. bigeniculata* and four *N. neesiana* populations). Numbers at the top of each panel are the broad sense heritability (*H*^2^) and the coefficient of variation (CV; expressed as a percentage of the mean) of the trait across clone lines (see methods). For species names, *Aubi* = *Austrostipa bigeniculata* and *Nane* = *Nassella neesiana*. For site names, Braid = Braidwood, Bung = Bungendore, Nanima = Nanima, and Wood = Woodstock (see Figure 1).

LMM analysis indicated that, under Phase I (ambient) common garden conditions, species (*Nassella vs*. *Austrostipa*), collection site and planting (original *vs*. replanted) were by far the strongest predictors of plant growth, reproduction and stature (Table 2). Although significant (*p* < 0.05) species × site interactions were present in all models, the width (Wid), volume (Vol) and stature (HWR) of tussocks were largely determined by species differences, with the overall larger size of *N. neesiana* indicating more rapid lateral growth and tussock expansion. With the exception of the Woodstock population (species × site interaction *p* < 0.05), *N. neesiana* tussocks were also more prostrate than those of *A. bigeniculata* (lower HWR; Table 2). *N. neesiana* produced many fewer flowering stems than *A. bigeniculata* (χ^2^ = 151.7, *p* < 0.001) during April (average = 0.04 *vs*. 0.64 stems dm^−2^), although species differences did depend significantly, but less strongly, on site (species × site interaction χ^2^ = 12.9, *p* < 0.01). Species differences in leaf elongation (LER) and biomass accumulation (BAR) depended more strongly on collection site (Table 2, Figure 5), with Woodstock *N. neesiana* exhibiting exceptionally rapid growth.

Replanted ramets tended to be smaller, to grow more slowly, and to have lower reproduction than original plants. Spatial (quadrant and quadrant position) predictors were also significant in some models (Table 2); in particular, plants growing on the inside of quadrants tended to be smaller and less prostrate than those located along the quadrant edge, and those in NE and NW quadrants grew more slowly than those in SW and SW quadrants. However, these effects tended to be much weaker than the species- and site-level differences previously described.

No clear clinal patterns were observed in any variable (Table 2) although leaf elongation rate in *A. bigeniculata* increased slightly with distance west of the collection site (Figure 5a). Variation in LER and BAR (Figure 5) was greater among *N. neesiana* populations, mainly reflecting the rapid growth of *N. neesiana* plants from Woodstock (Table 2; Figure 5a,b). Within-population (among clone line) variability (coefficient of variation of clone line means) in LER and BAR was generally similar in both species although the Woodstock *N. neesiana* population had reduced variation in both traits(Figure 5). Broad sense heritability (*H*^2^) was lower in LER than in BAR and tended to be lower in *N. neesiana* than *A. bigeniculata*. In *N. neesiana H*^2^ of both variables was lower in the range-edge Braidwood and Woodstock populations than in the range-core Nanima and Bungendore populations (Figure 5), but in *A. bigeniculata* this pattern was only observed for BAR in the Woodstock population (Figure 5a).

**Figure 6:**
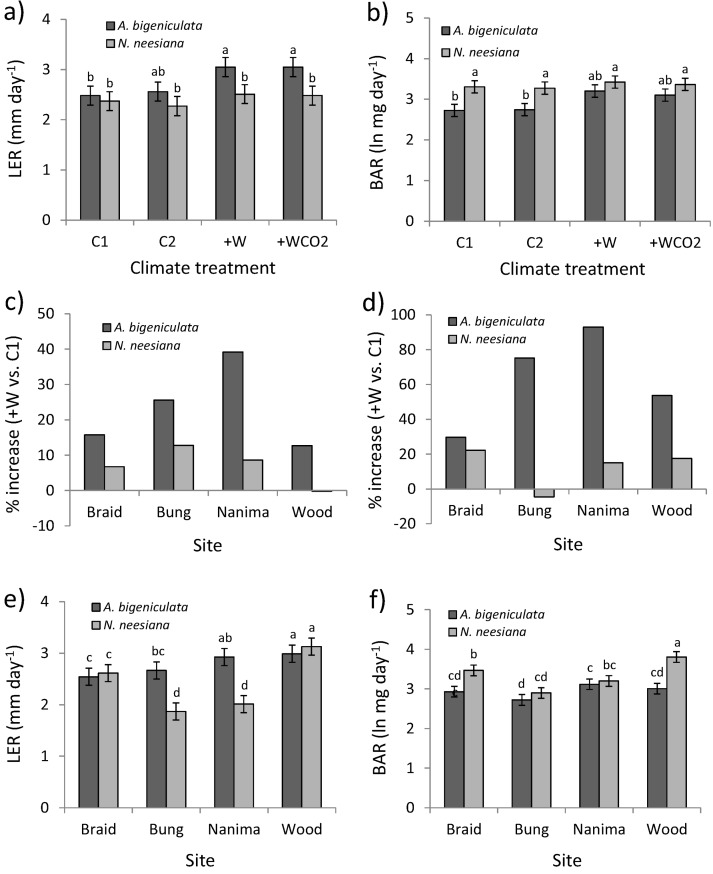
Leaf and biomass growth during Phase II of the experiment (June 2011). (**a**) Estimated leaf elongation rate (LER) (±1 SE) across climate treatments. (**b**) Estimated biomass accumulation (BAR) (±1 SE) across climate treatments. (**c**) Percentage increase in LER between warming (+W) and control (C1) treatments for each of the study populations. (**d**) Percentage increase (untransformed data) in BAR between warming (+W) and control (C1) for each of the study populations. (**e**) and (**f**) Estimated mean (±1 SE) LER and BAR across the study populations. Means sharing the same letter do not differ significantly at the 0.05 level. Climate treatment and site acronyms are as in Figure 3, Figure 5.

During Phase II species-level differences were again highly significant (*p* < 0.001; Table 3); overall *A. bigeniculata* exhibited more rapid leaf elongation (Figure 6a) but slower biomass accumulation (Figure 6b) than *N. neesiana*. However, differences depended on treatment (interaction *p* < 0.01; Table 3). For LER, species differences were only significant in +W and +WCO_2_ treatments (Figure 6a), since increased leaf growth due to warming (*i.e.*, +W treatment *vs*. C1 treatment) was much more pronounced in *A. bigeniculata* (12%–40%) than in *N. neesiana* (0%–13%; Figure 6c). Indeed, differences among treatments were significant in *A. bigeniculata* (*p* < 0.01) but not in *N. neesiana* (*p* = 0.52). For BAR, *A. bigeniculata* was more responsive to warming than *N. neesiana*, with treatment differences being significant only in *A. bigeniculata* (*p* < 0.01 *vs*. *p* = 0.77). Interspecific differences in BAR were greatest in control plots (C1 and C2; Figure 6b), due to the lack of response in *N. neesiana* to warming (Figure 6d); indeed, means of the two species in warmed treatments (+W and +WCO_2_) did not differ (Figure 6b). Species differences also depended strongly on collection site (interaction *p* < 0.001; Table 3). Leaf elongation was more rapid in *A. bigeniculata* than *N. neesiana* collected from Bungendore and Nanima (Figure 6e), while biomass accumulation was greater for *N. neesiana* than *A. bigeniculata* populations collected from the Braidwood and Woodstock sites (Figure 6f). There was again a trend towards increased leaf growth in *A. bigeniculata* populations collected from drier, warmer sites (Figure 6e), and variation among populations (sites) was higher in *N. neesiana* for both LER and BAR (Figure 6e,f).

**Figure 7:**
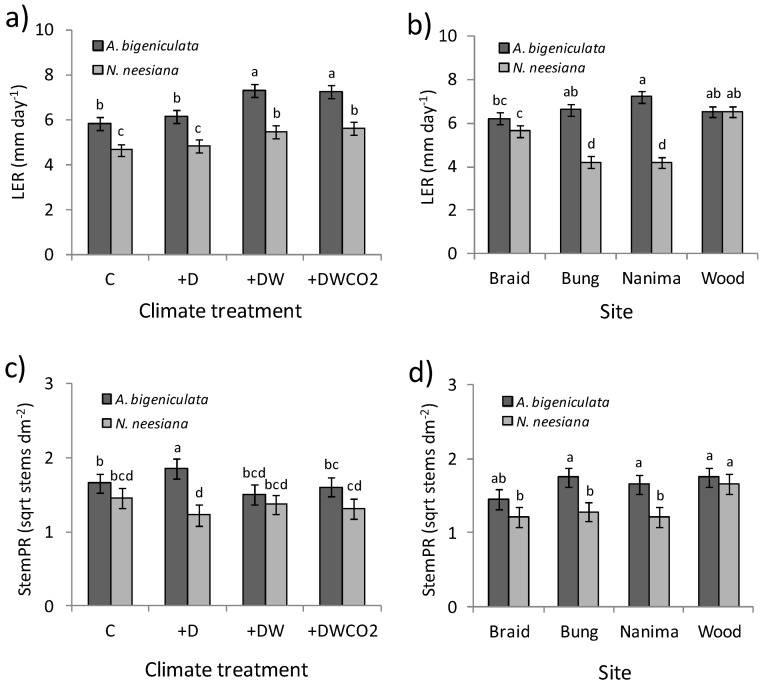
Growth of *A. bigeniculata* and *N. neesiana* during Phase III of the experiment. (**a**) Leaf elongation (LER) across climate treatments during August 2011. (**b**) LER across study populations during August 2011. (**c**) Stem production (StemPR) across climate treatments in November 2011. (**d**) StemPR across study populations in November 2011. Data are estimated means ± 1 standard error; means sharing the same letter do not differ significantly at the 0.05 level. Climate treatment and site acronyms are as in Figure 6.

During Phase III of the experiment drought was imposed and soil water declined (Figure 4). Early in this period (August), rain exclusion had no impact on leaf elongation (LER) (c.f., C and +D climate treatments, Figure 7a), but for both species growth was greater in warmed (+DW and +DWCO_2_) treatments than in the control. The increase in growth was greater in *A. bigeniculata* (23–25%) than in *N. neesiana* (18–20%), and while significant (*p* < 0.05), the associated treatment × species interaction term was weak relative to the main effects of species, site and climate treatment (Table 3). There was no evidence that CO_2_ enrichment altered leaf elongation rate in either species (c.f., +DW *vs*. +DWCO_2_ treatments; Figure 7a). The cline in LER across *A. bigeniculata* populations observed in June was weaker in August (Figure 7b), but differences among *N. neesiana* populations exhibited a similar pattern and there was again a much higher level of interpopulation variation than in *A. bigeniculata* (Figure 7b).

By November 2011, following an extended period of extremely low soil water, significant treatment × species interactions were observed in three variables: stem production rate (StemPR), maximum flowering stem length (MaxSL), and floret production rate (FlorPR) (Table 3). There was a strong overall pattern towards higher stem production in *A. bigeniculata* across treatments (main species effect *p* < 0.001; Table 3), although a significant (*p* < 0.05) post-hoc adjusted species difference was evident only in only in the drought (+D) climate treatment (Figure 7c). Stem production in *A. bigeniculata* exceeded that of *N. neesiana* only in Bungendore and Nanima populations (Figure 7d), resulting in a significant (*p* < 0.05) species × site interaction term for this variable (Table 3).

**Figure 8:**
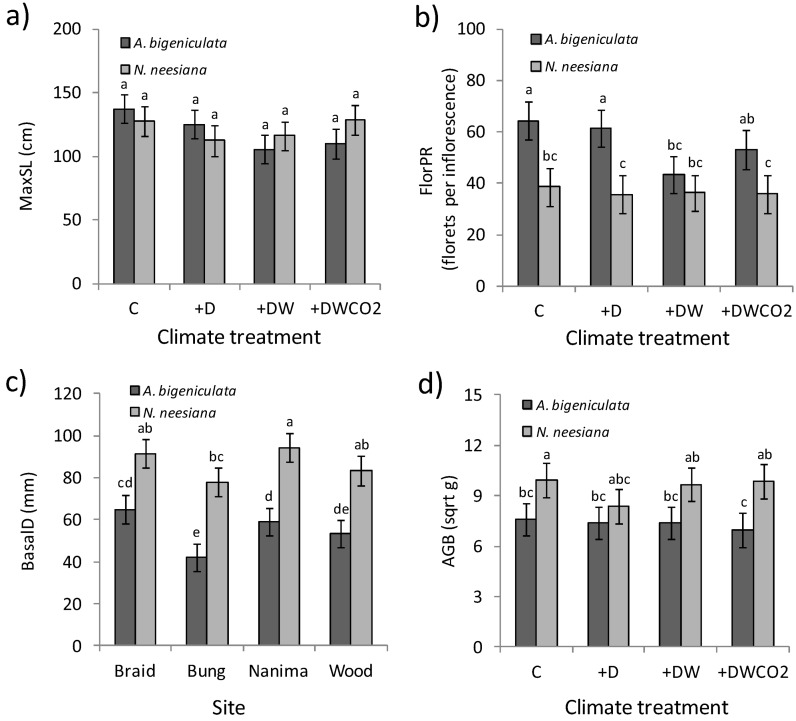
Reproduction and growth in *A. bigeniculata* and *N. neesiana* during Phase III of the experiment. (**a**) Maximum length of flowering stems (MaxSL) across climate treatments. (**b**) Floret production rate (FlorPR) across climate treatments. (**c**) Tussock basal diameter (BasalD) across study collection sites. (**d**) Above ground biomass (AGB) across climate treatments. Data are estimated means ± 1 standard error; means sharing the same letter do not differ significantly at the 0.05 level. In panel (a) estimated means differed at the 0.10 level (see text). Climate treatment and site acronyms are as in Figure 6.

The linear mixed model for MaxSL (Table 3) indicated that the two study species again responded differently to the range of climate treatments, albeit weakly (*p* < 0.05), with stem length influenced by the combination of drought and atmospheric warming only in *A. bigeniculata*. While *A. bigeniculata* treatment means differed only marginally (e.g., C *vs*. +DW mean difference *p* = 0.07; Figure 8a), overall differences among treatments were significant (*p* = 0.03), with stem length being ~20% lower in +DW and +DWCO_2_ treatments compared with the control. A similar pattern was observed for floret production, which, in *A. bigeniculata*, was significant reduced in the +DW treatment compared with the control (Figure 8b). Interestingly, drought (+D treatment) appeared to have no direct influence on floret production (Figure 8b). There was little evidence that atmospheric CO_2_ enrichment mitigated the effects of drought and heat stress observed in *A. bigeniculata*, with the possible exception of floret production (Figure 8b). In contrast to *A. bigeniculata*, *N. neesiana* did not differ significantly in stem length, stem production and floret production across any climate treatments (Figure 7c, Figure 8a,b; Table 3).

Other variables related to plant size and biomass (BasalD, AGB) also exhibited strong interspecific differences (Table 3), with *N. neesiana* tussocks being 62% wider and having 32% more biomass than those of *A. bigeniculata* (Figure 8c,d). These species-level differences tended to be similar across sites (e.g., Figure 8c) and treatments (Figure 8d), consistent with the very strong main effect of species and weaker site × species and treatment × species effects in the associated linear mixed models (Table 3). Tussock expansion (BasalE) in *N. neesiana* during Phases II–III of the experiment was more than double that of *A. bigeniculata* (Table 3).

## 4. Discussion

### 4.1. Drought, Climate and Adaptation in A. bigeniculata and N. neesiana

Despite being closely related stipoid grasses, *N. neesiana* and *A. bigeniculata* responded differently to atmospheric warming and soil drought. The observed patterns of change, however, provide little or no support for the hypothesis (H1) that NIPS advantage should be highest under more favorable growing conditions. Indeed, our data indicate that the NIPS *N. neesiana* is, if anything, less affected by thermal and hydrological change than the native species *A. bigeniculata*. This pattern was especially evident in the responses of leaf elongation and biomass accumulation to warming during autumn and early winter, which show that amelioration of cold temperatures most advantaged *A. bigeniculata* over *N. neesiana*. Indeed, species differences for these variables depended entirely on the presence or absence of atmospheric warming (Figure 6a,b).

There was also no evidence that *N. neesiana* growth and reproduction disproportionately declined under stressful (drought) conditions (c.f., [27]). Indeed, reproductive fitness (floret production) declined more in plots subjected to warming and drying (+DW treatment, Figure 8b) in *A. bigeniculata* than in *N. neesiana*, and similar trends occurred in maximum flowering stem length (Figure 8a) and (to a lesser extent) flowering stem production (Figure 7b). Whether these patterns reflect greater plasticity in resource allocation or phenology of *N. neesiana* under drought conditions (e.g., [68]) remains to be determined, but we did observe that the shift towards earlier flowering in drought treatments (+D, +DW and +DWCO_2_) was greater in *N. neesiana* (6.8 days) than *A. bigeniculata* (3.8 days; data in preparation). This may have helped generate the drought-related NIPS advantage in reproduction observed in *N neesiana* during the experiment. On the other hand, the much greater rate of autumn (April) flowering in *A. bigeniculata* is suggestive of a more facultative seed production in response to water availability, and indeed in southern NSW flowering in *N. neesiana* tends to be more tightly restricted to the spring and early summer. It must be remembered, however, that *N. neesiana* also produces cleistogamous stem and basal seeds, which may provide additional reproductive flexibility that supplements variation in timing and magnitude of panicle seed production [59]. This might mitigate any fitness consequences associated with the autumn flowering differential observed during our study.

However, while *A. bigeniculata* exhibited larger responses to warming and drought, *N. neesiana* still outperformed *A. bigeniculata* overall. By the end of the common garden experiment, *N. neesiana* tussocks were significantly larger than those of *A. bigeniculata*, and these differences increased through winter and spring. By the end of the experiment, mean basal diameter of *N. neesiana* tussocks was ~60% larger than that of *A. bigeniculata*, which alone doubled their relative potential reproductive fitness, at least in terms of total plant floret production. These differences probably arose primarily when water availability was adequate for growth, since in both species severe water stress developed only towards the end of the experiment. However, enhanced drought tolerance or water use efficiency [69] may also have contributed to the observed size differences.

The overall NIPS advantage observed in *N. neesiana* is not entirely unexpected since its invasiveness is thought to stem, as in many other weedy species [26,70], from its rapid growth rate and competitive ability [54]. The rapid biomass accumulation and prostrate growth habit observed in our study support the view that *N. neesiana* is highly competitive for light and other resources in grasslands, perhaps in an asymmetric manner in which initial differences in resource capture rates arising from size inequality are magnified by positive feedback between growth and resource acquisition [71,72,73]. It is also possible that other factors such as the presence of disease in sympatric native grass populations (for example in *Austrostipa*; [74]) and release from specialist pathogens (the enemy release hypothesis; [75]) may in part explain the competitive superiority of *N. neesiana* in mixed species stands and its ability to displace even dominant native species [58].

Collectively, our data suggest that overall NIPS advantage in *N. neesiana* is associated with rapid tussock expansion and increased plant basal area when soil water is available for growth. Since flowering stem production is related to overall plant size, this increases total reproductive potential if water availability reaches critical levels during spring drought. It appears that winter warming is likely to reduce this NIPS advantage but increasingly severe spring droughts may increase it. However, it is important to point out several caveats to this conclusion. First, we only simulated brief, acute drought, and, during spring, warming was unavoidably confounded with reduced soil water. Consequently, we cannot rule out variation in NIPS advantage in *N. neesiana* associated with the timing of warming and drought. Second, more protracted annual or multi-year drought may reduce the reproductive advantage observed in *N. neesiana* by more strongly limiting plant size. Third, the impacts of drought and atmospheric warming on other critical demographic variables (e.g., seedling recruitment, adult plant survival; [40]) may influence overall population fitness more than the growth and reproductive variables investigated in this study. Finally, variation in soil fertility may critically influence the ability of both species to exploit favorable climatic conditions and persist during drought. Answering these questions may resolve why *A. bigeniculata* appears to currently grow in warmer and drier areas of NSW than *N. neesiana* (Figure 1).

### 4.2. Warming, Elevated Atmospheric CO_2_, and Drought Severity

The specific climatic regimes applied in this experiment were intended to quantify the impacts of extreme drought on plant populations and to determine whether these impacts are likely to be exacerbated by future warming but ameliorated by rising atmospheric carbon dioxide concentrations (hypothesis H2). These are plausible expectations because elevated temperatures are known to increase drought severity [76,77] while elevated [CO_2_] increases plant water use efficiency [78,79] and generally reduces evapotranspiration [80].

As discussed above, measurements made at the end of the experiment indicate that soil drought significantly influenced plant growth and reproductive output. However, while we found evidence that additional spring warmth increases the impact of drought on some reproductive traits (e.g., floret production in *A. bigeniculata*), for all other variables, differences between drought and drought + warming treatments were not significant, and so we cannot be certain that warmer atmospheric temperatures will necessarily reduce general plant fitness via increased drought severity. There was also no support for the hypothesis that elevated [CO_2_] ameliorates drought severity; CO_2_ enrichment generated no obvious additive effects on plant growth nor significantly increased soil water content at any time during the experiment. We can therefore say little concerning the relative impact of CO_2_ enrichment of native and NIPS (c.f., [15,16]), except that neither appeared responsive to elevated [CO_2_] in this experiment.

There are several possible explanations for these results. First, surface evapotranspiration is not a direct function of plant stomatal conductance, but involves complex interplay between rainfall, radiative forcing, and the physiological and growth responses of plants to climate change, elevated [CO_2_], and soil nutrient status [81,82,83,84]. Indeed, elevated [CO_2_] can simultaneously reduce evapotranspiration by reducing plant stomatal conductance but increase it by enhancing plant growth (the CO_2_ fertilization effect; [83]). Not surprisingly, predicted changes in soil water balance in response to climate change vary across studies and display marked regional differences (c.f., [79,81,84,85]), and multi-factor manipulation experiments often show antagonistic or non-additive effects of temperature and [CO_2_] on plant growth and phenology ([86]; reviewed in [87]). Similarly complex processes may also have been operating in our experiment. For example, we observed that winter warming increased plant size, which in turn led to greater soil shading in spring when temperatures were higher. This could have mitigated increased evapotranspiration caused by warmer temperatures.

It is also possible that experimental conditions were not sensitive enough for the detection of subtle CO_2_-driven effects. While mostly close to the target range, [CO_2_] was variable and influenced by wind speed [66] and there were periods when CO_2_ flow was turned off. As noted above, compared with some other studies (e.g., [88]) the duration of warming and CO_2_ enrichment in our experiment was relatively short, and plant responses to both factors can change over time [84]. Perhaps we would have observed greater effects of warming and CO_2_ enrichment under the more chronic drought conditions which often characterize the Australian climate [89].

Finally, minor topoedaphic variability can have profound effects on plant populations during drought [40] and heterogeneity in soil structure and composition among plots may have generated significant variability in soil water availability that obscured treatment effects. Indeed, we did see differences in the behavior of soil water in plots during the common garden phase (I) of the experiment. Although plots were surrounded to a depth of 60 cm with black plastic to minimize water exchange with the surrounding soil, it is probable than greater isolation and homogenization of soil profiles would have increased our ability to detect the impact of warming and CO_2_ enrichment on soil water availability. Of course, this would also have limited our ability to extend experimental inference to true field conditions, since in nature topoedaphic variability is ubiquitous.

The points discussed above highlight the fact that simulating drought and interpreting impacts on plant fitness under field conditions is an exceptionally difficult exercise. Untangling the links between warming and plant performance under low soil water availability is one particular challenge. If soil water reserves exist prior to rainfall interception then the onset of soil drought is not instantaneous, and warming may initially relax thermal constraints but then magnify soil water deficits later. Indeed, such patterns may even occur over short timeframes in response to rainfall events which generate fluctuating soil water availability. Such patterns are likely to occur in nature and pose a major logistical challenge to reductionist experimentation.

Second, surprising declines in soil water may occur in ambient control plots, even when rainfall is high. For example, we found that soil water content frequently declined as much or more in control plots which received average rainfall as in drought-affected plots (Figure 4). This apparent tendency for loss of soil water to be positively related to soil water availability reduced differences among treatments. Finally, decisions concerning which traits to select for quantification are critical. For example, both of our study species flowered earlier in drought and warming treatments (data in preparation), which is classic drought avoidance behavior. In such cases, other traits such as drought survival and recovery may be under stronger selection pressure than those associated with growth and reproduction, leading to incorrect inferences about overall population fitness under climate change.

### 4.3. Genetic Diversity and Evolutionary Adaptive Potential in N. neesiana and A. bigeniculata

Our results provide mixed support for the notion that *N. neesiana* may have an evolutionary advantage over *A. bigeniculata* under climate change. First, intrapopulation-level quantitative variation for leaf and biomass growth traits was broadly similar in all *A. bigeniculata* and *N. neesiana* populations, although there was a trend for Woodstock *Nassella* populations to exhibit reduced variability compared with sympatric populations of *Austrostipa* (c.f., Figure 5a,b). Broad sense heritability of these traits was also lower in the two range-edge (Braidwood and Woodstock) *Nassella* populations (Figure 5), although it was also relatively low in the Woodstock *A. bigeniculata* population. This pattern likely reflects the relatively recent spread of *N. neesiana* to the Woodstock and Braidwood sites, probably from a very restricted number of founder propagules. Reduced genetic variation is frequently observed in range-edge plant populations [90,91], especially if isolated or founded by small numbers of propagules [92,93] and may reduce their evolutionary potential when placed under selection pressure. On the other hand, reduced within-population genetic diversity is also widely observed among selfing plant species [94,95], and both of the study species appear to produce a large proportion of selfed seed under field conditions.

In contrast, variation among *N. neesiana* populations tended to be considerably greater than among *A. bigeniculata* populations, which largely, but not solely, reflected the distinctive pattern of growth and reproduction in the Woodstock *N. neesiana* population. Having a history of multiple introductions is known to be a major source of genetic variation in many NIPS [96], and this is probably the case for *N. neesiana* in Australia. However, as noted previously, there is some uncertainty surrounding the taxonomic affinity of some *Nassella* genotypes in Australia, and we cannot rule out the possibility that multiple taxonomic entities have been introduced. If they are present, their ability to outcross and hybridize is also unknown. Nevertheless, increased interpopulation-level diversity is likely to favor the NIPS *N. neesiana* under climate change by increasing opportunities for both hybridization and the matching of extant genotypes with future climate regimes.

We also found little evidence for past climatic adaptation among populations of either *A. bigeniculata* or *N. neesiana*, apart from a weak clinal increase in leaf elongation rates with aridity among *A. bigeniculata* populations (Figure 5a, Figure 6a). Plant growth rates often tend to be negatively correlated with resource scarcity [24,97,98], and tradeoffs exist between growth rate and tolerance to abiotic stress [99,100], so this pattern is at least mechanistically plausible. Nonetheless, while not unique [92,101], the lack of population differentiation over such a large climatic gradient appears to be somewhat unusual among populations of native and exotic species generally (e.g., [20,22,23,102]). In the case of *N. neesiana*, this may reflect insufficient time for selection to have occurred, but why *A. bigeniculata* populations appear so similar across their range is more difficult to explain. Perhaps local adaptation has been prevented by ongoing migration of genetic material from core to peripheral populations [33,35,91], or perhaps phenotypic plasticity plays a more pivotal role than adaptive differentiation in generating reproductive assurance across the species’ range [21], especially given the highly variable climate experienced in southeastern Australia [89]. Quantification of the extent of local adaptation in native Australian plant species is consequently an area worthy of future research.

## 5. Conclusions

Our data provides some support for the basic premise that nonindigenous plant species (NIPS) are likely to outperform native species under future climate regimes. However, we found no evidence that NIPS advantage is greater under more favorable growing conditions, in this case winter warming, nor reduced during drought. Indeed, the NIPS *N. neesiana* appeared to be less responsive to climatic and edaphic variation than the similar native species *A. bigeniculata*, with the overall fitness advantage of *N. neesiana* being associated with more rapid tussock expansion and to a lesser extent by maintenance of reproductive output during drought. The evolutionary potential of *N. neesiana* appears to be restricted by low trait heritability and genetic diversity, especially in range-edge populations, although high levels of genetic diversity among populations may increase the potential for climate matching and the development of novel genotypes via hybridization. We conclude that NIPS advantage under climate change may be limited by a lack of responsiveness to key climatic drivers and reduced genetic variability in range-edge populations.
